# Supplementary material for: The Prevalence, Management and Impact of Dysmenorrhea on Medical Students’ Lives—A Multicenter Study
Source: Healthcare (Basel). 2022 Jan 14;10(1):157. doi: 10.3390/healthcare10010157 (PMC8775858; doi:10.3390/healthcare10010157)
Supplement: Supplementary file 1 [file healthcare-10-00157-s001.zip › healthcare-1512202-supplementary.pdf]

healthcare

MDPI

The impact of dysmenorrhea on the quality of life of medical students

I. GENERAL CHARACTERISTICS

1. How old are you? ...
2. The university center where you study:
   - -Bucharest
   - -Cluj-Napoca
   - -Craiova
   - -Targu Mures
   - -Timisoara
3. How many births have you had? ...
4. How many abortions have you had? ...
5. At what age did menarche (first menstruation) occur? ....
6. How are your menstrual cycles?
   - -Regular (21-35 days)
   - -Irregular (<21 days or> 35 days).
7. How long does menstruation last? (bleeding days)
   - -1-3 days
   - -3-5 days
   - -> 5 days.
8. How do you assess the amount of blood lost during menstruation?
   - -Normal bleeding (7-10 pads used)
   - -Abundant bleeding (> 10 pads used)
   - -Reduced bleeding quantitatively (<7 pads used)
9. Do you use internal tampons during menstruation?
   - -Yes, during every period
   - -I've never used it
   - -Occasionally
10. You have a personal history of: (you can choose one or more options)
    - -Gynecological surgeries
    - -Genital malformations
    - -Recurrent vaginal candidiasis
    - -Repeated urinary tract infections
    - -No variant of the above
11. You have been diagnosed with: (you can choose one or more variants)
    - -Endometriosis
    - -Polycystic ovary syndrome
    - -Pelvic inflammatory disease
    - -An autoimmune disease
    - -No variant of the above
12. Have you been treated for Chlamydia trachomatis or Neisseria Gonorrhoeae?
    - -Yes
    - -No
13. What was the last result of the Babeș-Papanicolau test?
    - -Normal
    - -Inflammation
    - -Cellular abnormalities (ASCUS, L-SIL, H-SIL, AGC)
    - -Cancer
    - -I didn't take this test
14. What kind of sex life do you have?

-Activate
-Inactive.

15. What contraceptive method do you use? (you can choose one or more variants)
    -Preservative
    -Oral contraceptives
    -Intrauterine contraceptive device
    -Coitus interruptus
    -Another method
    -Not applicable
16. Do you have pain during sexual intercourse (dyspareunia)?
    -Yes, over 50% of sexual intercourse
    -Yes, less than 50% of sexual intercourse
    -No
    -Not applicable
17. Do you have sexual intercourse when you are menstruating?
    -Never
    -Occasionally
    -At each period
18. Do you do regular physical activity?
    -Yes (at least one hour twice a week)
    -No
19. Do you have a healthy diet?
    -Yes (diet rich in fruits and vegetables, low in fat)
    -No
20. Are you a smoker?
    -Yes
    -Former smoker
    -I've never smoked
21. What foods or drinks do you usually eat? (you can choose one or more variants)
    -Alcoholic beverages (at least 30g of alcohol in a week - the equivalent of about 300ml of wine or 3 bottles of 330ml beer)
    -Coffee (250 ml of coffee at least 3 times a week)
    -Coca Cola or Pepsi (250 ml at least 3 times a week)
    -Black tea (300 ml at least 3 times a week)
    -Chocolate (30g at least 3 times a week)
    -Spicy foods
    -No variant of the above
22. How many hours do you normally sleep each night?
    -<6 hours
    -6-9 hours
    -> 9 o'clock
23. Do you have premenstrual syndrome (the appearance of mood changes a few days before menstruation, breast tenderness, change in appetite, fatigue, bloating, acne)?
    -Yes, at each menstrual cycle
    -Occasional
    -No
24. Do you have a family history of dysmenorrhea (painful menstruation)?
    -Yes
    -No
25. Do you have dysmenorrhea (painful menstruation)?
    -Yes
    -No (questionnaire closes)

II.DYSMENORRHEA CHARACTERISTICS

If you have dysmenorrhea, answer the following questions:

26. When did dysmenorrhea occur?
    - -From the first period
    - -At an interval of 6-24 months after the first menstruation
    - -Less than a year
    - -From 1-3 years
    - -From 4 years or more
27. When does dysmenorrhea start?
    - -On the first day of menstruation
    - -The day before menstruation
    - -More than two days before menstruation
28. How often does menstrual pain occur?
    - -At <35% of menstrual cycles
    - -At 35-70% of menstrual cycles
    - -At > 70% of menstrual cycles
    - -At each period
29. Where is the most intense pain located?
    - -At the pelvis and lower abdomen
    - -At the lumbar level
    - -At the thighs

30.Where does the radiation radiate?
   - -At the lumbar level
   - -At the thighs
   - -Both at the lumbar level and at the thighs
   - -Another location
   - -Does not irradiate

31. How intense is the pain?
    - -Easy
    - -Moderate
    - -Severe

32.Give a pain score on a scale of 1 to 10, where 1 is no pain and 10 is unbearable pain: ...

33.Do you think that menstrual pain is more intense when you have a stressful period?
   - -Yes
   - -No

34. Has the intensity of the pain decreased since the beginning of sexual life?
    - -Yes
    - -No
    - -Not applicable

35.Did the intensity of the pain decrease after the first birth?
   - -Yes
   - -No
   - -Not applicable

36. How long does the pain last?
    - -Few hours
    - -One day
    - -Two days or more
37. What methods do you use to reduce pain?
    - -Pharmacological methods

-Non-pharmacological methods (for example: massage of painful regions, application of liquids or hot objects on the abdomen)
-Both
-I use nothing to reduce pain

38. What medicines do you use to reduce pain?
-Non-steroidal anti-inflammatory drugs
-Antispastic
-Both
-Another type of medicine
-I don't use drugs

39. When do you start taking medication? (you can choose one or more variants)
-Every time before the pain appears
-Before the pain only appears if you have scheduled events or activities
-With the appearance of pain regardless of its intensity
-When the pain becomes unbearable
-I don't use drugs

40. Do you think that medicines help to reduce pain?
-Yes, every time
-Yes, occasionally
-No
-I don't use drugs

41. If you take medication when the pain occurs or when the pain is unbearable, how intense is the pain? (If not applicable, go to the next question) Rate the pain on a scale of 1 to 10, where 1 is no pain and 10 is unbearable pain: ....

42.If you are taking medication before the onset of pain, do you feel that the intensity of the pain that is about to occur is lower?
-Yes, if I take medication at least 3 days before menstruation
-Yes, regardless of the number of days of administration
-No
-Not applicable

43.If you are taking medication long enough before your period, how intense is the pain? (If not applicable, go to the next question) Rate the pain on a scale of 1 to 10, where 1 is no pain and 10 is unbearable pain: ....

44.Does the administration of medicines help you to carry out your daily activities?
-Yes
-No
-Not applicable

45.What non-pharmacological methods do you use to reduce menstrual pain? (you can choose one or more variants)
-Do not use non-pharmacological methods to reduce pain
-Consumption of sweets
-Walk
-Sleep
-Massage of painful regions
-Application of liquids or hot objects on the abdomen
-Using warm tampons
-Other non-pharmacological methods

46. Along with pain, what other symptoms occur during menstruation? (you can choose one or more variants)
-Nausea

-Vomiting
-Headache
-Diarrhea
-Sweating
-Loss of appetite for food
-Insomnia
-Fatigue
-Feeling dizzy
-Polyuria
-Arthralgia
-Agitation / irritability
-Only the pain appears

47. What is the most annoying symptom that accompanies pain?
-Nausea
-Vomiting
-Headache
-Diarrhea
-Sweating
-Loss of appetite for food
-Insomnia
-Fatigue
-Feeling dizzy
-Polyuria
-Arthralgia
-Agitation / irritability
-Not applicable

III. THE IMPACT OF DYMENOREA ON THE QUALITY OF LIFE

48. Do you think that dysmenorrhea affects your quality of life?
-Yes
-No
49.What characteristic of menstrual pain do you consider to be the most important for altering the quality of life?
-The duration
-Intensity
-convenient

50. Do you plan your vacations or trips according to your menstrual period?
-Yes
-No

51. Do you change your clothing style when you are menstruating (do you prefer to wear pants)?
-Yes
-No

52.When you have menstrual cramps: (you can choose one or more options)
-You feel more agitated or more nervous
-You feel more tired
-You have a higher level of stress
-You have less energy for daily activities
-You can't have a normal diet
-You cannot perform normal physical activities
-No variant of the above

53.Consider that dysmenorrhea affects you in a negative way: (you can choose one or more variants)
-social life

-relationships with family members
-couple relationship
-relationships with friends
-school work
-No variant of the above

54. How are family relationships affected when menstrual cramps occur? (you can choose one or more variants)
-Consider that you have conflicts with family members due to your condition
-Try to isolate yourself from family members
-You care less about family duties
-Consider that family members do not understand your suffering and are not with you
-No variant of the above

55. Are couple activities affected when menstrual cramps occur?
-Yes, most of the time
-Not
-Occasional

56. When you are menstruating, do you feel increased affection and attention from your life partner?
-Yes
-No

57. How are relationships with friends affected when menstrual cramps occur? (you can choose one or more variants)
-Avoid meeting friends
-Discuss less with your friends
-No variant of the above

58. How is school performance affected when menstrual cramps occur? (you can choose one or more variants)
-You can't concentrate on classes
-You cannot accumulate the same amount of information
-You failed the exams
-Consider that your exam grade is influenced by your condition
-Individual study is affected
-None of the above

59. Usually, how many days do you have to miss classes?
-One day
-2-3 days
-More than 3 days
-I'm not missing classes
